# A Multidisciplinary Evaluation of Three-Dimensional Polycaprolactone Bioactive Glass Scaffolds for Bone Tissue Engineering Purposes

**DOI:** 10.3390/ma17102413

**Published:** 2024-05-17

**Authors:** Gregorio Marchiori, Devis Bellucci, Alessandro Gambardella, Mauro Petretta, Matteo Berni, Marco Boi, Brunella Grigolo, Gianluca Giavaresi, Nicola Baldini, Valeria Cannillo, Carola Cavallo

**Affiliations:** 1 Scienze e Tecnologie Chirurgiche, IRCCS Istituto Ortopedico Rizzoli, 40136 Bologna, Italy; gregorio.marchiori@ior.it (G.M.); gianluca.giavaresi@ior.it (G.G.); 2 Department of Engineering “Enzo Ferrari”, University of Modena and Reggio Emilia, 41125 Modena, Italy; devis.bellucci@unimore.it (D.B.); valeria.cannillo@unimore.it (V.C.); 3 REGENHU SA, 1690 Villaz-St-Pierre, Switzerland; 4 Laboratorio di Tecnologia Medica, IRCCS Istituto Ortopedico Rizzoli, 40136 Bologna, Italy; matteo.berni@ior.it; 5 Scienze e Tecnologie Biomediche e Nanobiotecnologie, IRCCS Istituto Ortopedico Rizzoli, 40136 Bologna, Italy; marco.boi@ior.it (M.B.); nicola.baldini@ior.it (N.B.); 6 Laboratorio RAMSES, IRCCS Istituto Ortopedico Rizzoli, 40136 Bologna, Italy; brunella.grigolo@ior.it (B.G.); carola.cavallo@ior.it (C.C.); 7 Department of Biomedical and Neuromotor Sciences, University of Bologna, 40126 Bologna, Italy

**Keywords:** PCL, bioactive glasses, therapeutic ions, magnesium, composite scaffolds, human bone-marrow-derived mesenchymal stem cells, tissue engineering, bone

## Abstract

In the development of bone graft substitutes, a fundamental step is the use of scaffolds with adequate composition and architecture capable of providing support in regenerative processes both on the tissue scale, where adequate resistance to mechanical stress is required, as well as at the cellular level where compliant chemical–physical and mechanical properties can promote cellular activity. In this study, based on a previous optimization study of this group, the potential of a three-dimensional construct based on polycaprolactone (PCL) and a novel biocompatible Mg- and Sr-containing glass named BGMS10 was explored. Fourier-transform infrared spectroscopy and scanning electron microscopy showed the inclusion of BGMS10 in the scaffold structure. Mesenchymal stem cells cultured on both PCL and PCL-BGMS10 showed similar tendencies in terms of osteogenic differentiation; however, no significant differences were found between the two scaffold types. This circumstance can be explained via X-ray microtomography and atomic force microscopy analyses, which correlated the spatial distribution of the BGMS10 within the bulk with the elastic properties and topography at the cell scale. In conclusion, our study highlights the importance of multidisciplinary approaches to understand the relationship between design parameters, material properties, and cellular response in polymer composites, which is crucial for the development and design of scaffolds for bone regeneration.

## 1. Introduction

Bone is a mineralized connective tissue exerting key functions in the human body, enabling locomotion, supporting and protecting soft tissues, and acting as a storage of mineralized components [1]. Bone is able to respond and adapt its structure and composition to the complex scenario of loading at which it is normally exposed thanks to the dynamic remodeling potential guaranteed by the interaction between the extracellular matrix (ECM) and the tissue-specific cells, namely osteoblasts and osteoclasts [2,3]. The dynamic remodeling of bone can be impaired by traumatic events or by degenerative pathologies, the last increasing in rate due to the prolonged life expectancy [4], potentially causing defects in bones unable to self-heal. In treating such conditions, bone tissue engineering approaches based on scaffolds emerged as a promising solution, allowing the development of three-dimensional (3D) substitutes with features resembling native tissue [5]. A suitable 3D scaffold should promote the proliferation and differentiation of bone cells [6], i.e., osteoinduction, and provide load-bearing capacity [7], with the purpose of replicating bone structure, shape, and overall function [8]. Given such a challenging scenario, polymers represent an eligible option, allowing to tailor and optimize simultaneously the biological, structural, and mechanical features of 3D constructs, which can be used alone or synergistically with non-polymeric materials [9].

In light of these considerations, different formulations of synthetic polymers have been employed in bone tissue engineering (BTE) approaches, such as poly(methyl methacrylate), polyethylene, polypropylene, polyurethane, poly(-ethylene terephthalate), poly ether ketone, and polysulfone. The most commonly used in BTE applications are represented by aliphatic polyesters such as poly(lactic acid)(PLA), poly(glycolic acid)(PGA), poly(caprolactone) (PCL), and their copolymers), due to their favorable biocompatibility and biodegradability properties combined with suitable mechanical properties and processability by means of different scaffold fabrication techniques [10].

Among them, PCL is one of the most employed in bone tissue engineering for the fabrication of 3D scaffolds due to its unique properties. A Food and Drug Administration (FDA) approved polyester, PCL possesses good biocompatibility and mechanical properties and is easily processable by extrusion-based 3D printing due to its high thermal stability and low melting temperature (around 60 °C). Moreover, compared to other aliphatic polyesters such as PLA, PLGA, and their blends, PCL is characterized by a slower degradation rate, which provides a more adequate time for bone remodeling and a lesser number of acidic breakdown products [11].

Despite the low cost and the easy processing, the employment of PCL for addressing bone tissue engineering purposes entails some drawbacks—i.e., hydrophobicity, low bioactivity, slow degradation rate, and poor mechanical properties—therefore, it needs improvements for challenging biomedical applications [5]. A common approach to improve the features of PCL scaffolds is to develop constructs combining this polymer with other components [12,13,14]. In this regard, scaffolds composed of a mixture of PCL and ceramics/bioactive glass succeeded in providing higher bioactivity and rate of biodegradability over pure PCL constructs [15]. The employment of these composite materials can favor the differentiation of bone cells, thanks to the release of ions and an increase in the mechanical response (stiffness), therefore accelerating the formation of bone tissue [16,17,18,19,20]. Besides the employed materials, i.e., chemistry, the performance of the 3D scaffold strictly depends on its architecture and surface physical properties. For this reason, additive manufacturing technologies have recently emerged as a promising tool for bone tissue engineering, given their unique capability of processing a wide range of different materials, including polymers, metals, ceramics, and composites, to fabricate structures with control over the internal architecture down to the microscale [21,22].

The main features that define the internal architecture of a 3D scaffold are pore size and porosity [23]. This aspect is of paramount importance since it determines (i) migration and proliferation of cells, (ii) diffusion of nutrients essential for cell viability, and (iii) vascularization of the scaffold [4]. Architectures with interconnected pores of around 300–800 μm in size proved to be eligible in addressing bone-tissue engineering purposes, therefore allowing to reach significant levels of tissue growth and regeneration [22]. The surface topography of the scaffold is also a crucial factor that must be considered. More in detail, topographic features—e.g., specific roughness and presence of micro/nanopatterns- proved to enhance protein adsorption, cell adhesion, proliferation, and osteoinductivity [4,24,25,26,27,28]. Last, surface mechanical properties, e.g., elastic or Young’s modulus, have an important role in determining the cellular fate [29]. Therefore, the suitability of a 3D scaffold in responding to a specific application should be investigated by different approaches involving multiple spatial scales and chemical and the assessment of various chemical and physical parameters. In this study, we present a multidisciplinary framework evaluating the eligibility of a 3D composite PCL-based composite scaffold for bone tissue engineering purposes. The construct examined here represents the third step in the development and optimization of a PCL-based scaffold, which was first designed [30] and then tested for cell viability and cytotoxicity [20]. It was produced by combining PCL and a bioglass (BG) formulation recently developed named BGMS10. This material, owing to its composition containing MgO, SrO, and a significantly lower content of alkali metals compared to standard commercial references—such as the well-known 45S5 or S53P4—is capable of combining a high crystallization temperature with excellent biological performance and in vitro bioactivity [31,32]. In this work, Fourier-Transform Infrared Spectrophotometry (FTIR) was applied to evaluate the composition and structure of both PCL and BGMS10 before printing, and of printed fibers of PCL and of the composite, that from now on will be named PCL-BGMS10. Scanning electron microscopy (SEM) along with energy dispersive X-ray analysis (EDX) was then carried out to investigate the morphological features of PCL and PCL-BGMS10 fibers. Then, the ability of both pure PCL and PCL-BGMS10 scaffolds to promote osteogenic differentiation of human bone marrow mesenchymal stem cells (BM-MSCs) was investigated by molecular biology, evaluating gene expression of specific osteogenic markers on days 7, 14, and 21. X-ray Micro-Computed Tomography (MicroCT) allowed performing a volumetric investigation of PCL and PCL-BGMS10 composite scaffolds, in particular evaluating (i) their architecture, (ii) the size and distribution of BGMS10 particles and, moreover, (iii) eventual cellular deposit after culture. Finally, Atomic Force Microscopy (AFM) was employed to investigate the surface topography and stiffness of the scaffold fibers at the cell scale, with the aim to reveal whether the addition of BGMS10 has changed the near-surface morphological and mechanical characteristics of the scaffold. 

## 2. Materials and Methods

### 2.1. Preparation

#### 2.1.1. Preparation of Glass Powders

The BGMS10 bioactive glass (composition in mol%: 2.3 Na_2_O; 2.3 K_2_O; 25.6 CaO; 10.0 MgO; 10.0 SrO; 2.6 P_2_O_5_; 47.2 SiO_2_) used in conjunction with PCL for scaffold production was manufactured using a conventional melting procedure. The powdered raw materials (SiO_2_, Ca_3_(PO_4)2_, Na_2_CO_3_, CaCO_3_, K_2_CO_3_, SrCO_3_, MgCO_3_, all reagents grade, by Carlo Erba Reagenti, Rodano-Milano, Italy), accurately weighed and mixed, were melted in a platinum crucible at 1450 °C for an hour. The following thermal cycle was employed to melt the starting powders: from room temperature to 1100 °C at a rate of 10 °C/min, followed by a one-hour thermal hold at 1100 °C to allow for the decomposition of carbonate raw materials, and then a subsequent ramp at 10 °C/min until reaching the final temperature of 1450 °C. The molten material was quenched in water at room temperature to obtain a frit, which was later dried for 12 h at 110 °C. Finally, the frit was dry-milled for 40 min in an alumina jar with alumina balls, and the resulting powder was manually sieved to achieve a final particle size <63 µm [20].

#### 2.1.2. Preparation of the Poly(ε-Caprolactone)/Bioactive Glass Composite

The PCL-BGMS10 composite was obtained by dissolving PCL pellets (MW = 80,000, Sigma Aldrich, St. Louis, MO, USA) in chloroform (Sigma Aldrich) under magnetic stirring and gradually adding BGMS10 particles to the solution until a 50/50 wt% ratio was reached. To avoid clustering and enhance particle dispersion within the composite, the solution was first stirred for 24 h at room temperature and then sonicated for 30 min before precipitation into an excess of ethanol. The final composites were obtained after solvent evaporation by air-drying for 24 h in a chemical hood and loaded into the hot-melt extrusion printhead after pelletization.

#### 2.1.3. 3D Scaffold Fabrication

**Figure 1:**
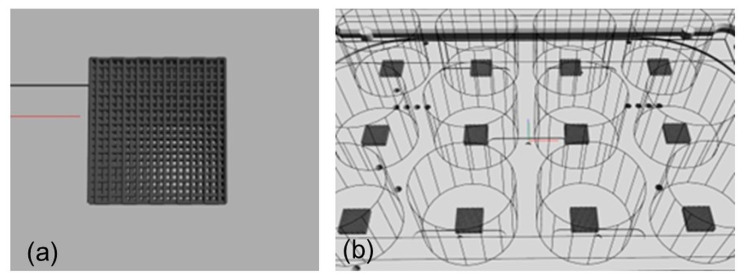
(**a**) design of the PCL-BGMS10 scaffolds. The red line is the axis origin while black line indicates landmarks on the building plate (**b**) Automated printing in 12-well plates. Blue-red lines are the axis origin, while black dots indicate reference points on the building plate.

Pure PCL and PCL-BGMS10 scaffolds were fabricated as follows. First, 5 × 5 × 3 mm^3^ structures were modeled by means of BioCAD software version 1.1 (RegenHU, Villaz-St-Pierre, Switzerland). The following design parameters were used for the scaffold internal microarchitecture: fiber diameter of 300 μm, interfilament distance (center-center of two adjacent fibers) of 600 μm, 0°/90° grid deposition pattern. Additionally, an offset equal to half-interfilament size (300 µm) for every repeating unit (0°/90°/0° off/90° off) to guarantee an increased surface for seeded cell adhesion (Figure 1a). The layer height was set to 100 μm to provide proper stacking and avoid collapsing effects due to the relaxation of the molten fibers. The process was designed to be performed directly within 12-well culture plates (Euroclone, Milan, Italy). A parallel printing mode was chosen, to give time to each deposited layer to cool down before the next layer was stacked and thus minimize fiber collapse and enhance shape fidelity. A representative image of the final result is provided in Figure 1b. 

The high-temperature pneumatic extrusion printhead of a 3D Discovery platform (RegenHU, Villaz-St-Pierre, Switzerland) was employed for the hot-melt extrusion process. Construct sterility was ensured by performing the process in a Class II biosafety cabinet, in which the 3D Discovery platform is embedded.

Composite pellets were loaded into a stainless-steel cartridge and mounted into the printhead. To increase the melt homogeneity, the pellets were kept at the printing temperature for 30 min before starting the printing process. A conical, flow-optimized stainless-steel nozzle with an inner diameter of 300 µm was used for the process. The printing was performed on a heated building plate to overcome issues related to warping effects and enhance the deposition accuracy.

**Figure 2:**
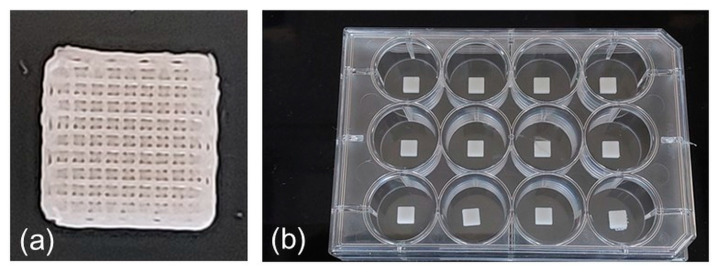
Macroscopic pictures of the fabricated scaffolds. (**a**) 12-well plate. (**b**) Single scaffold close-up.

A total of 50 cubic scaffolds were fabricated for both the composite formulation and pure PCL control for the morphological and biological characterization tests. Single filaments were also extruded using the same printing parameters and conditions and collected for AFM characterizations. The optimal printing parameters to process the PCL and PCL-BGMS10 composite formulations, identified in the testing phase, are reported in Table 1. Macroscopic photographs of the fabricated scaffolds are presented in Figure 2.

### 2.2. Fourier Transform Infrared Spectroscopy (FTIR) Analysis

Macroscopically, FTIR spectra were collected on the PCL, PCL-BGMS10 pellets, and BGMS10 powder using a PerkinElmer Spectrum 2 (Waltham, MA, USA) in the Attenuated Total Reflection (ATR) mode equipped with a diamond crystal (sample area of 7 × 3 mm^2^), with a resolution of 4 cm^−1^, a scan step of 0.5 cm^−1^ and 32 accumulations. The wavelength range was set from 4000 to 400 cm^−1^. Microscopic compositional analysis was carried out onto printed fibers using the PerkinElmer Spotlight 200i microscope coupled to a Spectrum 2 instrument and equipped with an ATR of germanium crystal. The following acquisition parameters were used: resolution 4 cm^−1^, scan step 0.5, and 32 scans. Spectra were acquired on a sample area of 50 × 50 µm^2^ areas, and the ATR probe of the microscope applied a load of 25% onto printed fibers. The analyses were performed on monolayers printed directly onto microscope slides and not on 5 × 5 × 3 mm^3^ scaffolds, similar to the next paragraph.

### 2.3. Scanning Electron Microscopy (SEM) Analysis

The surface of the PCL and PCL/BGMS10 scaffolds was observed in a scanning electron microscope (SEM—ESEM Quanta 2000, FEI Co., Eindhoven, The Netherlands) equipped with X-ray energy dispersion spectroscopy (EDS, Inca, Oxford Instruments, Abingdon, UK). Prior to observation, the scaffolds were sputter-coated with Au.

### 2.4. MSC Seeding and Osteogenic Differentiation

MSCs, derived from bone marrow left over from a previous study approved by the Ethics Committee of Istituto Ortopedico Rizzoli (n. 0021750), were anonymized and used in the present study. Cells were thawed, expanded for two passages, and seeded onto PCL and PCL-BGMS10, a number of 7.0 × 10^5^ on each scaffold. MSCs were allowed to adhere to the scaffolds for at least 30 min at room temperature, and then α-MEM 15% FBS (ThermoFisher Scientific, Waltham, MA, USA) was added for 24 h. The following day, the medium was changed to osteogenic medium consisting of α-MEM containing 15% FBS, 10^−7^ M dexamethasone (Sigma, St. Louis, MO, USA), 75 μg/mL ascorbate-2 phosphate (Sigma), and 0.01 mM β-glycerolphosphate (Sigma). Constructs were cultured for up to 21 days, and molecular biological analyses were performed on 7, 14, and 21 days.

### 2.5. mRNAs Expression by Real-Time PCR

Cells obtained from both PCL and PCL-BGMS10 and differentiated into osteogenic lineage were analyzed by real-time PCR to investigate the expression of specific osteogenic markers such as bone sialoprotein (BSP), osteocalcin (OC), osteopontin (OP), alkaline phosphatase (ALP), RUNX family transcription factor 2 (RUNX), and the transcription factor osterix (OSX). To this aim, RNA was extracted from both PCL and PCL-BGMS10 constructs using TRIzol reagent (Invitrogen, Waltham, MA, USA) according to the manufacturer’s instructions. After treatment with DNase I (DNA-free Kit; Ambion, Life Technologies, Carlsbad, CA, USA) and RNA quantification using a Nanodrop^®^ spectrophotometer (EuroClone S.p.a., Pero, Italy), 0.5 μg of RNA were reverse transcribed using MuLV reverse transcriptase (Thermo Fisher Scientific, Waltham, MA, USA). PCR primers for the selected genes and for the housekeeping gene glyceraldehyde-3-phosphate dehydrogenase (GAPDH) used as an internal control are listed in Table 2. Real-time PCR was performed with the following protocol: initial activation at 95 °C for 10 min, amplification for 45 cycles at 95 °C for 5 s and 60 °C for 20 s, in a LightCycler instrument (Roche Molecular Biochemicals, Indianapolis, IN, USA) using SYBR Premix Ex Taq (Takara, Clontech Laboratories, Mountain View, CA, USA). mRNA levels were calculated for each target gene and normalized using the reference gene GAPDH according to the formula 2^−ΔCt^.

### 2.6. Statistical Analysis of Gene Expression

The distribution of gene expression values for each type of scaffold and experimental time was assessed via the Kolmogorov–Smirnov test. Consequently, and focusing on the individual type of scaffold, Kruskal–Wallis test was applied to investigate statistical differences among different experimental times. Dunn test with Bonferroni correction was used as a post hoc. Statistical difference in terms of gene expression between pure PCL and PCL-BGMS10 scaffolds was investigated—at each experimental time—by the Wilcoxon rank test. The significance threshold was set to 0.05. Statistical analyses were performed by using R software (version 4.1.2, Wien, Austria) and Matlab (MATLAB 2022b, MathWorks, Natick, MA, USA).

### 2.7. Microtomography (MicroCT) Analysis

MicroCT analysis involved two samples for each composition, i.e., PCL and PCL-BGMS10, in dry condition and without cell seeding, in order to obtain information on the architecture of 3D printed constructs along with the BGMS10 distribution.

Furthermore, MicroCT analysis was performed on two samples for each scaffold type and for the shortest and longest seeding time, i.e., 7 and 21 days, after fixing and drying the samples, in order to reveal possible cellular deposits. MicroCT scanning and image reconstruction protocols were specific for each scaffold type; indeed, a common protocol would yield suboptimal results for both cases.

For PCL, acquisition of the sample images was performed in a MicroCT apparatus (Skyscan 1172, Bruker, Kontich, Belgium) with the following scan parameters: pixel size (nominal spatial resolution) of 5 µm, no scan filter, source tube voltage of 40 kV, scan orbit of 180°, rotation step of 0.3°. Images were reconstructed by using a modified Feldkamp algorithm implemented in the Skyscan NRecon software (version 1.7.4.6) with the following reconstruction parameters: level 1 Gaussian smoothing, level 5 ring artifact correction, and no beam hardening. For PCL-BGMS10, acquisition of the sample images was performed in the Skyscan 1172 with the following scan parameters: pixel size (nominal spatial resolution) of 5 µm, 0.5 mm thick aluminum filter, source tube voltage of 70 kV, scan orbit of 360°, rotation step of 0.3°. Images were reconstructed in the Skyscan NRecon software with the following reconstruction parameters: no Gaussian smoothing, level 5 ring artifact correction, and 40% beam hardening correction.

Then, with the Skyscan CT-Analyser (CTAn) software (version 1.20.3.0), images were segmented to binary and analyzed morphometrically in an ad hoc selected region of interest (ROI). An ROI wrapping the scaffold was implemented to calculate fiber thickness, pore size, and total porosity. Moreover, for PCL-BGMS10, additional ROIs pertaining to the scaffold fibers were built up to analyze BGMS10 particle size and regional distribution. To separate the scaffold from the background, global thresholds were selected by visual matching with grayscale images: global threshold values were 55–255 in the PCL case and 66–255 in the PCL-BGMS10. To separate BGMS10 from PCL in the analysis of PCL-BGMS10, global thresholds were set to obtain a polymer-to-particles volume ratio of 70/30%, which corresponds to the designed 50/50 wt% ratio assuming a PCL density of 1.145 g/cm^3^ “https://www.xpolymers.it/pcl.html (accessed on 11 June 2023)” and a BG density of 2.7 g/cm^3^ [33]: global thresholds values for BGMS10 resulted 120–255. The reader may refer to the Supplementary Materials Section for Figures S1 and S2 mentioned in the following.

Regarding morphometric analysis, both 2D and 3D analyses of pore size were performed to highlight the difference in measuring it on a specific plane (2D, e.g., Figure S1) or integrating the various planes (3D). Moreover, 3D analysis calculated fiber thickness and total porosity.

Regarding BGMS10, the 3D analysis allowed us to obtain (i) on the global scaffold, the particles’ size distribution, (ii) on concentric ROIs (Figure S2), its dispersion (i.e., % of ROI volume) moving from fibers’ surface to fibers’ core.

Finally, looking for cellular deposits eventually visible in MicroCT images, dataset gray-scale histograms of seeded scaffolds were compared with those of as-printed scaffolds, both for PCL and PCL-BGMS10.

### 2.8. Atomic Force Microscopy (AFM) Analysis

**Figure 3:**
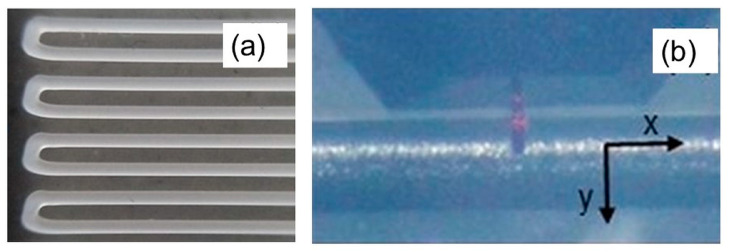
(**a**) Optical image of the microfibers deposited onto a glass slide and used for FTIR and AFM analyses. (**b**) Optical image showing the orientation of the microfiber with respect to the scan directions of the AFM measurement.

PCL and PCL-BGMS10 fibers were deposited onto glass slices to facilitate the AFM measurement (Figure 3a). An NT-MDT (Moscow, Russia) AFM system was used, equipped with an upright optical microscope which allows to properly adjust the position of the probe on the sample. The single microfiber alignment (along the *x*-axis) was set orthogonally to the slow scan direction (i.e., along the *y*-axis, Figure 3b). NSG10 cantilevers (NT-MDT, Moscow, Russia) with resonant frequency in the range 140–390 kHz and stiffness *k* = 10.4 N/m were used in tapping mode of operation for both topographies (resolution 512 × 512-pixel) and extraction of Young’s modulus via nanoindentation, i.e., acquisition of force curves according to a procedure described elsewhere [34]. Before and after images/curves acquisition, the tip integrity was checked via *z*-axis calibration on a TGS1 calibration grating (NT-MDT, Moscow, Russia; grid TGZ1 with height (21 ± 1) nm). According to the manufacturer, the tip apex is spherical with curvature radius R = (10 ± 2) nm. Several force curves acquired at equally spaced points along the *x* and *y*-axes within a given topographic region constitute a map of force curves. The maps shown here contain 60 × 24 = 1440 force curves each. Young’s modulus was calculated from each force curve by a contact model, which accounts for elastic deformation of the surface, assuming ideal conditions (negligible adhesion forces, isotropy, homogeneity) at each point of indentation [34].

## 3. Results

### 3.1. FTIR Analysis

**Figure 4:**
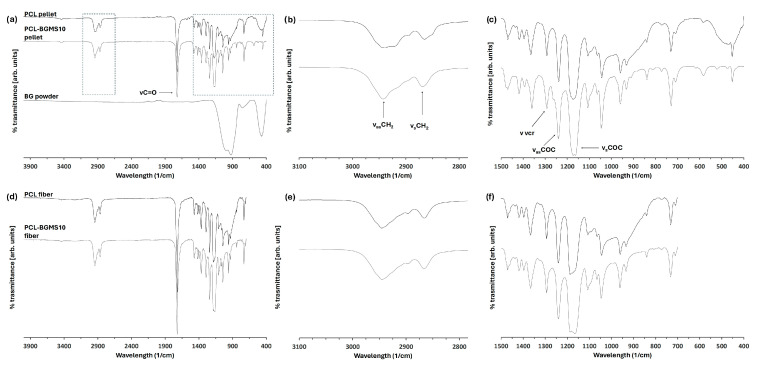
FTIR spectra of (**a**–**c**) precursor material: PCL-BGMS10 (black line) and PCL (charcoal line) pellets and BGMS10 powder (light gray line) and 4 (**d**–**f**) printed fibers of PCL-BGMS10 (black line) and PCL (charcoal line). In (**a**) for PCL pellets are reported the symmetric (υ_s_) and asymmetric (υ_as_) vibrations discussed in the manuscript. The dashed boxers zoom (**b**,**e**) the methylene asymmetric (υ_as_CH_2_) and symmetric (υ_s_CH_2_) stretchings between 3100 and 2800 cm^−1^ and (**c**,**f**) the bands related to the crystalline phase (υcr) and the asymmetric (υ_as_C-O-C) and symmetric (υ_s_C-O-C) ester stretching between 1500 and 400 cm^−1^.

In Figure 4a, a characterization of the PCL pellets is reported. The methylene asymmetric (υ_as_CH_2_) and symmetric (υ_s_CH_2_) stretchings were observed at 2942 and 2864 cm^−1^, respectively, while the carbonyl (υC = O) stretching band appeared at 1722 cm^−1^. A band at 1292 cm^−1^ related to the C-O and C-C stretching in the crystalline phase (υcr), and two bands at 1238 and 1160 cm^−1^ could be ascribed to the asymmetric (υ_as_C-O-C) and symmetric (υ_s_C-O-C) ester stretching, respectively [35].

The BGMS10 powder spectrum was optically transparent up to 1180 cm^−1^ and exhibited four intense bands at 987, 912, and 743 cm^−1^, probably related to O-Si-O stretching of the silicate group [35] and at 462 cm^−1^, possibly assigned to P-O bonds of PO_4_^3–^ group [36], or the O-Si-O bending of the silicate group [37,38].

Several changes in the PCL-BGMS10 spectra were noticeable when compared to the bands of the PCL pellets. Overlapping of the BGMS10 bands was observed in the wavelengths 1180–790 and 571–400 cm^−1^ (Figure 4c). In methylene region 2600–3000 cm^−1^, changes in the doublet of the υ_as_CH_2_ and υ_s_CH_2_ indicated conformational differences in the aliphatic chains related to amorphous and crystalline phases. Specifically, in crystalline PCL pellets, the υ_as_CH_2_ was sharper than the PCL-BGMS10 ones [39,40] (Figure 4b). The broader band observed in PCL-BGMS10 pellets indicated a reduction in the crystallinity of the PCL polymeric matrix.

Finally, a difference was found between pure PCL and PCL-BGMS10 pellets in the υsC-O-C. In the PCL pellets, two distinct peaks were noticeable at the wavelength of 1160 cm^−1^, while in the composite material, there was a single broadband that could be ascribed to the overlapping Si-O-Si group of bioactive glasses (Figure 4c). FTIR analysis was then performed on PCL and PCL-BGMS10 fibers using microscopy, which enabled the investigation of an area of 50 × 50 µm^2^. However, the microscopy configuration can only acquire vibrational spectra up to 700 cm^−1^, so the BGMS10 band at 462 cm^−1^ was not detected (Figure 4d). In both spectra, the peak positions and bandwidths did not vary compared to the PCL pellets. Once again, in PCL-BGMS10, the overlap of the BGMS10 bands at wavelengths 1180–790 and 571–400 cm^−1^ was detected. Regarding the methylene region 2600–3000 cm^−1^, both printed fibers exhibited bands similar to those of the PCL pellets (Figure 4e).

### 3.2. SEM Measurements

**Figure 5:**
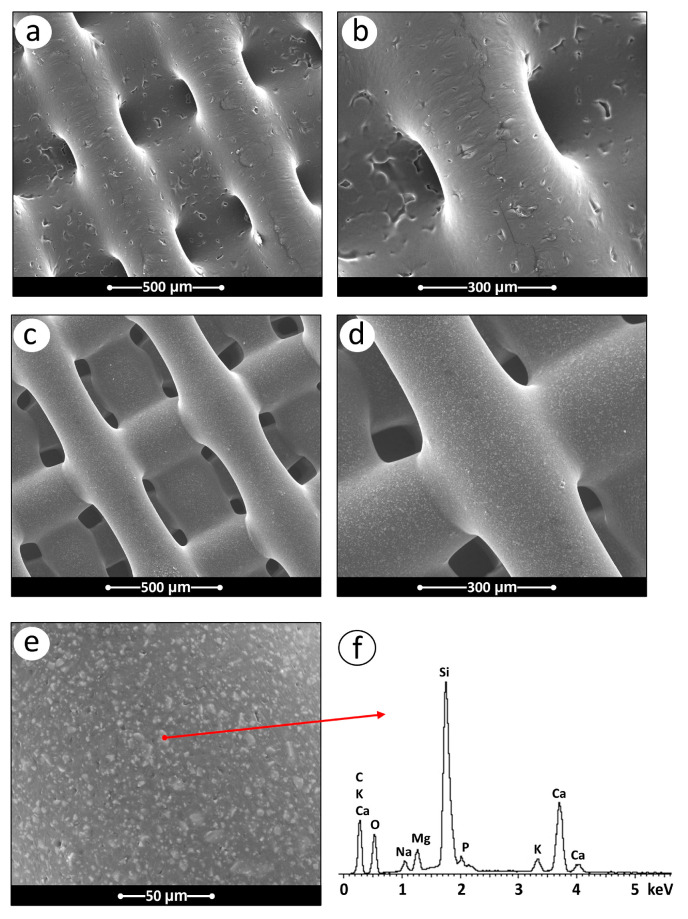
(**a**,**b**) Representative SEM micrographs of the PCL and (**c**–**e**) PCL/BGMS10 scaffolds. (**e**,**f**) Results of the X-ray microanalysis performed at the point reported in (**e**).

The SEM images of the PCL and PCL-BGMS10 scaffolds are reported in Figure 5, accompanied by the results of the X-ray microanalysis carried out on the PCL-BGMS10 sample. Compared to PCL (Figure 5a,b), the PCL-BGMS10 images show a more compact and homogeneous surface (Figure 5c,d), with micrometers and sub-micrometers-sized protrusions/islands uniformly distributed across the surface (see the zoomed-in image in Figure 5e). The microanalysis conducted on these particles distinctly reveals the presence of ions inherent in the glass composition (Figure 5f).

### 3.3. Gene Expression

**Figure 6:**
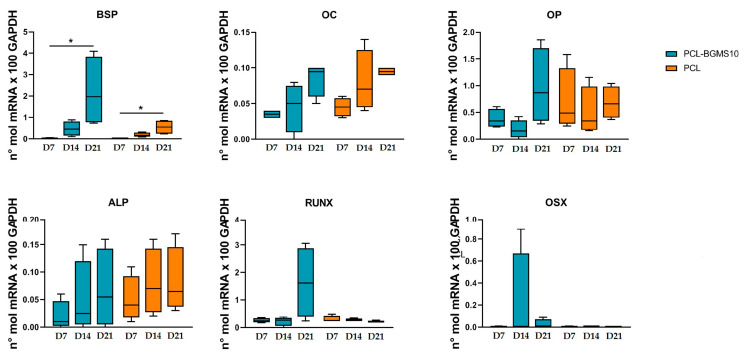
Tukey box plot showed gene expression at 7, 14, and 21 days of the principal osteogenic markers. Different patterns were used for different scaffolds: PCLBGMS10 blue, PCL orange. * *p* < 0.05.

The expression of BSP, OC, OP, ALP, RUNX, and OSX was evaluated by real-time PCR at 7, 14, and 21 days. MSCs grown on both PCL and PCL-BGMS10 showed the same trend, gradually increasing the expression of the above-mentioned osteogenic genes until day 21. In particular, the BSP gene level displayed a higher expression at day 21 compared to day 7 in both PCL and PCL-BG constructs (*p* < 0.05). Regarding the ability of the PCL-BGMS10 scaffold to induce a higher osteogenic differentiation compared to PCL, no significant differences were observed, although MSCs grown on PCL-BGMS10 displayed a higher gene expression of BSP and RUNX (Figure 6).

### 3.4. MicroCT Analysis

**Figure 7:**
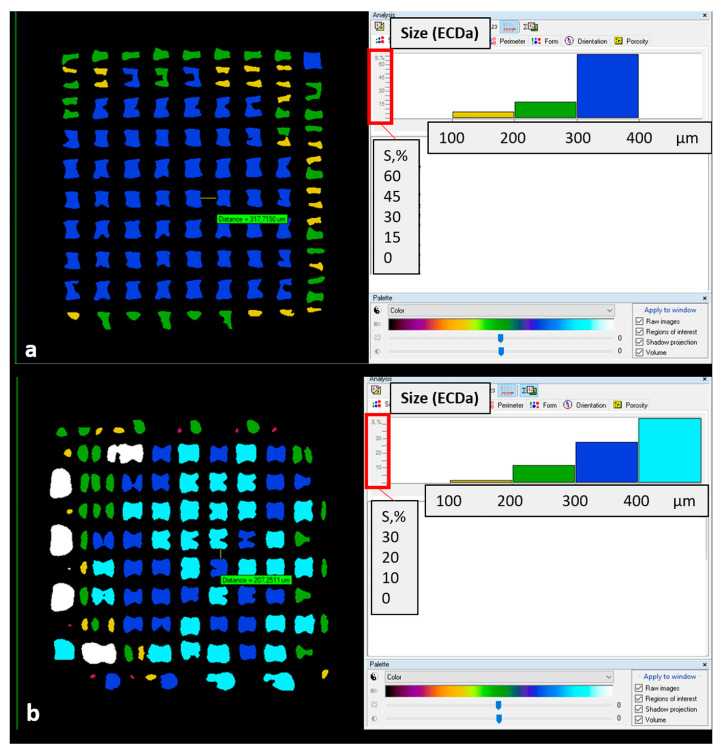
Transversal sections of (**a**) PCL scaffold and (**b**) PCL-BGMS10scaffold, mapping pore size (2D parameter “Size (ECDa)”) with colors and percentage on surface area (S,%) histograms.

**Figure 8:**
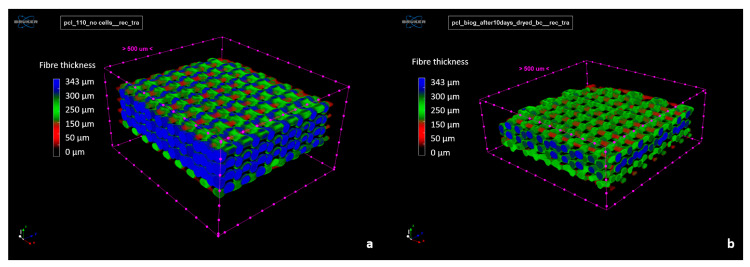
Fiber thickness mapping of a PCL (**a**) and PCL-BGMS10 (**b**) scaffold, 3D rendered by SkyScan CTVox software (version 3.3.1).

On specific transversal sections, pore size seemed close to the 300 µm nominal size for the PCL scaffold, even higher for the PCL-BGMS10 scaffold (Figure 7). Integrated 3D parameters—i.e., fiber thickness, pore size, and total porosity—resulted in (287 ± 10) µm, (100 ± 8) µm and (21 ± 6)% for PCL; (253 ± 11) µm, (205 ± 24) µm and (47 ± 4)% for PCL-BGMS10. Even if calculated only on two samples for each scaffold type, the PCL-BGMS10 scaffold seems far more porous and with thinner fibers (Figure 8) than the PCL scaffold. Porosity resulted in fully interconnected in both scaffold cases.

**Figure 9:**
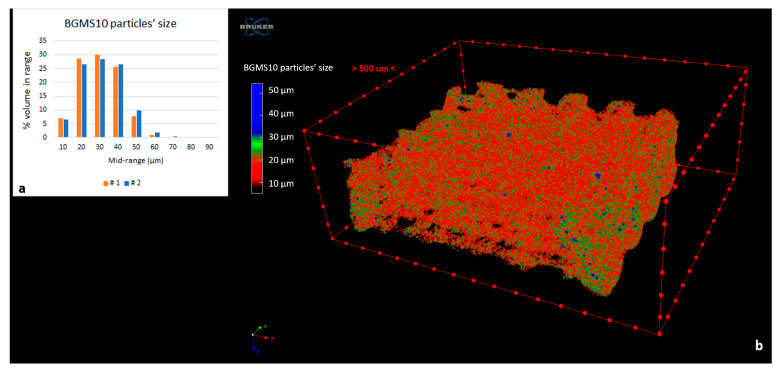
BGMS10 particles’ size (**a**) distribution of the two PCL-BGMS10 samples (#1 and #2) and (**b**) mapping of a sample, 3D rendered by SkyScan CTVox software. Eventual particles below 5 µm cannot be seen because of MicroCT spatial resolution.

**Figure 10:**
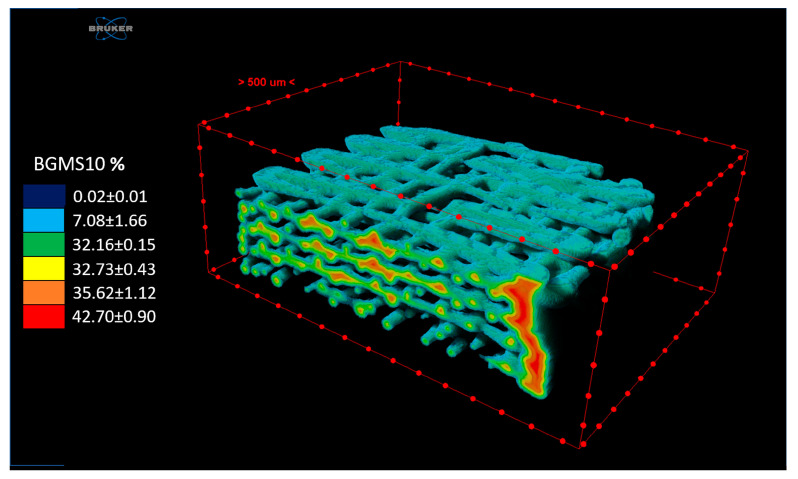
% of BGMS10 content expressed as (mean ± SD) of the two samples, in concentric ROIs mapped on a PCL-BGMS10 scaffold, 3D rendered by SkyScan CTVox software.

Regarding BGMS10 particles’ size distribution as imaged by MicroCT, it appeared centered on an equivalent sphere diameter of 30 µm, with the biggest granules showing a 60 µm diameter Figure 9). Moreover, BGMS10 percentage content increased from fiber surface to fiber core (Figure 10).

**Figure 11:**
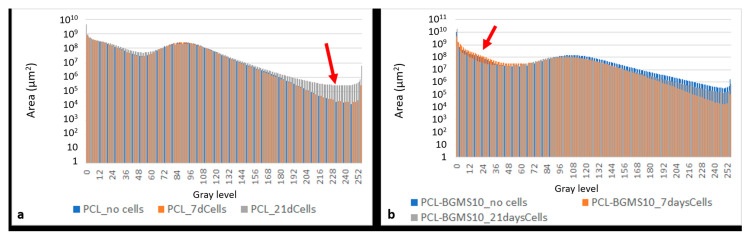
MicroCT image dataset gray-scale histograms (0–255 gray levels) showing (**a**) greater areas with high gray levels (180–255, red arrow) for a PCL scaffold harvested for 21 days (PCL_21dCells) respect to the baseline (PCL_no cells) and (**b**) greater areas with low gray levels (12–60, red arrow) for PCL-BGMS10 scaffolds harvested for 7/21 days (PCL-BGMS10_7dCells/PCL-BGMS10_21dCells) respect to the baseline (PCL-BGMS10 no cells).

**Figure 12:**
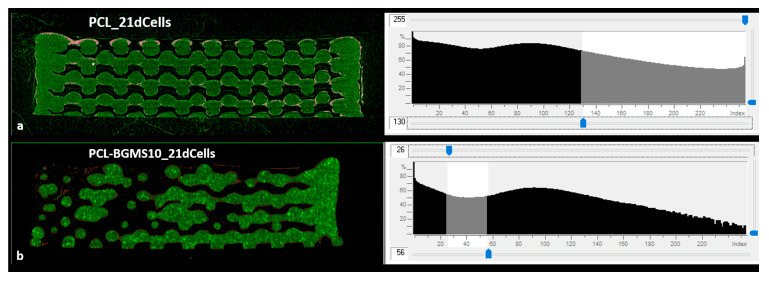
Sagittal cut of MicroCT image reconstruction (left) and corresponding gray-scale histogram (right) of (**a**) PCL scaffold harvested for 21 days (PCL_21dCells) and (**b**) PCL-BGMS10 scaffold harvested for 21 days (PCL-BGMS10_21dCells), showing possible cellular deposit (in pink or red in sagittal cut images, with relative gray levels highlighted in histograms) on the scaffold (in green in sagittal cut images, gray levels not highlighted in histograms) surfaces.

Finally, by a qualitative comparison with MicroCT image of baseline not seeded scaffolds, in seeded scaffolds, a major contribution at high gray levels for PCL (Figure 11a) and at low gray levels for PCL-BGMS10 scaffolds (Figure 11b) could correspond to a cellular deposit (Figure 12).

### 3.5. Atomic force Microscopy

**Figure 13:**
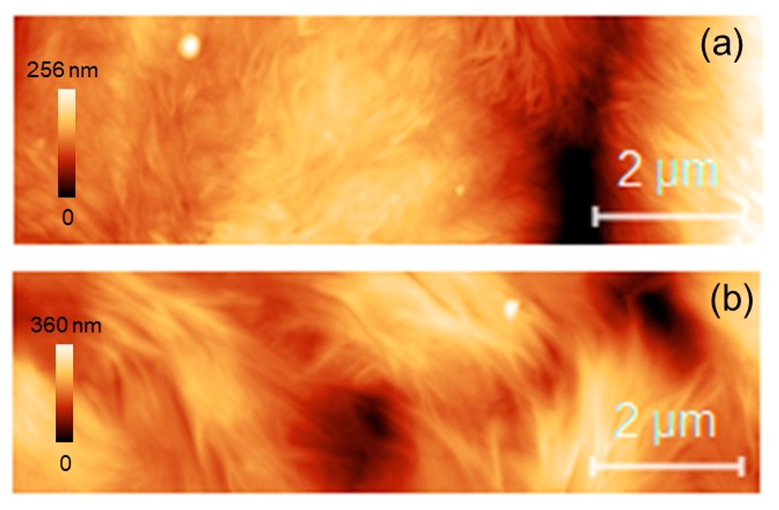
Topographic 10 µm × 4 µm images of (**a**) PCL and (**b**) PCL-BGMS10.

**Figure 14:**
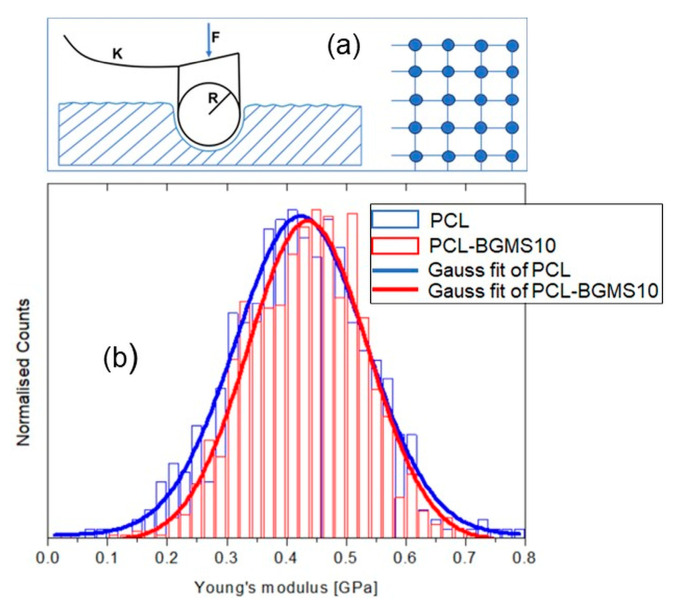
(**a**) Schematic of the AFM-based nanoindentation and (**b**) Statistical distribution of Young’s moduli extracted from 1440 nanoindentation points taken on the topographic regions of Figure 13a (blue bars) and Figure 13b (red bars) and the corresponding Gaussian fittings (software OriginPro version 7.5).

Representative 10 µm × 4 µm AFM topographies of both PCL (Figure 13a) and PCL-BGMS10 (Figure 13b) samples are reported. It is remarkable that both regions display the fiber-like nature of the polymer surface, as observed in a previous study of this group [29]. However, a significant increase in the peak-to-peak roughness from PCL to PCL-BGMS10 samples was also observed. The two regions displayed here were also investigated nanomechanically, i.e., a grid of nanoindentations was operated within the topographies after the image acquisition, according to the procedure described in Section 2.8. Previous findings established that an applied load of F ≈ 50 nN at each point of indentation could guarantee purely elastic deformation of the surface [29]. Thus, such load was maintained throughout the experiment on both fiber types. The spacing between the indents was 10,000 nm/60 ≈ 167 nm (Figure 14a). Hence, Young’s moduli distributions could be extracted from the indentations and reported (Figure 14b). Both the PCL (blue bars) and PCL-BGMS10 (red bars) distributions resulted in a Gaussian-like in shape (Shapiro–Wilk test with *p* = 0.05), with a central value of (0.42 ± 0.15) and (0.44 ± 0.15) GPa, respectively, hence reporting no remarkable differences between the mechanical response of the fibers.

## 4. Discussion

Compositional analysis performed by FTIR revealed the presence of bands corresponding to both PCL and BGMS10 vibrational levels in the composite material, considering both their pellet and monolayer printed forms.

Qualitatively, the FTIR results show that the addition of amorphous BGMS10 to PCL reduces the crystallinity of the composite, a circumstance favored by the fact that the preparation process of the composite pellets takes place at room temperature, thus most likely without the kinetic energy required to enhance the crystallinity of the polymer.

On the other hand, the observation of a similar degree of crystallinity between PCL and PCL-BGMS10 fibers may reasonably be ascribed to the fact that the extrusion takes place at 120 °C, followed by a slow cooling process. In this case, the available thermal energy could favor the reorganization of the polymer chains, increasing the recrystallization of the printed fiber.

Compared to PCL, on PCL-BGMS10 samples, SEM was capable of capturing morphological features that may reasonably correspond to localized and (sub) micrometers large regions where BGMS10 particles/aggregates protrude from the surface, hence being potentially accessible to cells by direct contact. This result is not contradictory with the observed MicroCT results, which highlighted BGMS10 segregation to the inner core of the scaffolds and reduction in the BGMS10 content from the center to the surface of the fiber (Figure 5 and Figure 6). This implies that BGMS10 particles/aggregates having a dimension below the spatial resolution of the MicroCT (5 µm) may have reached the scaffold’s surface; this scenario is compatible with the SEM findings of Figure 5e,f and with the interaction volume of the X-ray microanalysis, typically probing a few micrometers below the surface. On the tissue scale, PCL-BGMS10 scaffold architecture, as imaged by MicroCT, appeared less regular and more porous with respect to PCL, two factors that could limit cell exposure to the bioactive glass. On the other hand, in terms of osteogenic differentiation, MSCs grown on both PCL and PCL-BGMS10 showed similar trends, with all genes evaluated increasing from day 7 to day 21, demonstrating their ability to differentiate towards the osteogenic lineage. These results agree well with those obtained by MicroCT analysis (Figure 7 and Figure 8), which showed a deposition of the extracellular matrix on both types of scaffolds. However, our results did not show significant differences between the two scaffolds in terms of osteogenic gene expression. Reasonably, this indicates that there was no or little direct contact between cells and BGMS10 particles, which are most likely embedded into the polymeric matrix. Daskalakis et al. observed that the addition of BG to PCL scaffolds had no significant impact on the cell attachment process, suggesting a major role of micro-architecture on the biological performance of the scaffolds [19]. However, this study did not evaluate osteogenic differentiation. On the other hand, Kim and coauthors demonstrated a remarkable increase in osteogenic potential by incorporating a BG into the PCL scaffold at a concentration similar to our case. Independently on the specific BG formulations, this result could be attributed to the average size of the BG particles used (~2 µm), i.e., one order of magnitude smaller than ours. Probably, the smaller particle size allows better dispersion of the BG within the polymer matrix, enabling it to reach the surface and to affect cell differentiation within short (7 days) experimental times [41]. In our case, the experimental times scheduled herein (up to 21 days), although in line with previous studies on cell differentiation, did not allow for the degradation of the PCL and the consequent exposure of the BGMS10, so that the cells were only partially stimulated by the presence of this material. On the side, the slow degradation rates of PCL could represent an advantage in the long term, as this peculiar feature has proven to be favorable to bone remodeling in providing a structural guide to complete the regeneration process in critical-size defects [11]. Nevertheless, it could be suggested that a homogenous mixture of BG within the polymer is crucial to guarantee an effective cell-surface interaction.

The observations above are strongly supported by the AFM characterization. This technique was by far the most surface-sensitive sample characterization among those used in the present work due to its superior spatial resolution and capability of probing only the very first layers of the surface. First, the difference between the morphological features observed in PCL compared to PCL-BGMS10 may be, at least partially, explained by the change in the polymer structure and fiber alignment that normally arises from variations in composition and/or printing process [29]. Moreover, the slight peak-to-peak (or roughness) increase in correspondence of the BGMS10 is compatible with the SEM findings, i.e., with an overall increase in topographic complexity due to the protrusion of BGMS10 particles or aggregates. Finally, by looking at the nanoindentation, the shallow surface deformation did not show an increase in the surface stiffness due to the direct contact between the tip apex and the BGMS10 particles but only probed the mechanical characteristics of the polymeric layer that covers the particles in a homogenous fashion. As established previously on PCL within a similar experimental context, the penetration depth corresponding to an applied load of ≈50 nN is around 20 nm [29]. On the other hand, it is well known that bulk contributions to Young’s modulus are unimportant for penetration depths 10 s times smaller than the layer’s thickness [42]. Given that the BGSM10 particles are much stiffer than PCL, in the present case, we have to conclude that their contribution to the measured Young’s modulus was negligible (the average Young’s modulus did not change much from PCL and PCL-BGMS10); thus, BGMS10 particles are covered by a homogeneous polymer layer around 200 nm in thickness, or more. This agrees with the SEM-EDX findings and with the long degradation time of PCL. Moreover, the homogeneous distribution of moduli is compatible with the absence of clear patterns in the spatial distribution of moduli observed in PCL in similar experimental conditions, which concurs to the paucity of mechanical stimuli capable of inducing bone tissue integration and bioactivity [29,43]. This scenario agrees well with the FTIR results that showed no significant changes in the degree of crystallinity between the two scaffolds.

## 5. Conclusions

Three-dimensional scaffolds based on polycaprolactone reinforced with a new bioglass containing Mg and Sr for bone tissue engineering purposes were proposed. The optimization of the scaffolds’ design and processing was finalized in previous works. Here, the composite scaffolds were compared with pure polycaprolactone scaffolds and investigated by means of a multidisciplinary approach, including bone cell differentiation, scanning electron microscopy, Fourier-transform infrared analysis, micro-computed tomography, and atomic force microscopy. The little difference between pure polymer and composite scaffolds in their capability of inducing bone cell differentiation at 7 and 21 days of culture was ascribed to the presence of a homogeneous polymer layer that surrounds the bioglass particles, preventing direct contact with cultured cells at the scaffold’s surface. This conclusion agrees well with the tomographic analysis, which showed a diminution of bioglass from the inner core to the surface of the single scaffolds’ fiber and is in line with the compositional analyses that showed the presence of bioglass within a few microns from the surface.

## Figures and Tables

**Table 1:** Optimized printing parameters for PCL and PCL-BGMS10.

Parameter	PCL	PCL-BGMS10
Pressure (Bar)	5	5
Speed (mm/s)	4	4
Printhead Temperature (°C)	110	120
Collector Temperature (°C)	30	40
Start Delay Time (ms)	0	200

**Table 2:** List of primers used in real-time PCR.

RNA Template	Primer Sequences (5′-3′)	Annealing Temperature (°C)
GAPDH	5′-TGG TAT CGT GGA AGG ACT CAT GAC 3′-ATG CCA GTG AGC TTC CCG TTC AGC	60
BSP	5′-CAGTAGTGACTCATCCGAAG 3′-CATAGCCCAGTGTTGTAGCA	60
OC	5′-GCAGCGAGGTAGTGAAGA 3′-TCCTGAAAGCCGATGTGG	60
OP	5′-ATGATGGCCGAGGTGATAG 3′-GCTTTCCATGTGTGAGGTG	60
ALP	5′- GGAAGACACTCTGACCGT 3′- GCCCATTGCCATACAGGA	60
RUNX	5′- GGAATGCCTCTGCTGTTATG 3′- AGACGGTTATGGTCAAGGTG	60
OSX	5′-TGCTTGAGGAGGAAGTTCACTATG 3′-AAAGGTCACTGCCCACAGA	60

## Data Availability

Data are contained within the article and Supplementary Materials.

## Supplementary Materials

The following supporting information can be downloaded at https://www.mdpi.com/article/10.3390/ma17102413/s1. Figure S1: Cutting planes of the MicroCT image reconstruction of a PCL scaffold (a) and of a PCL-BGMS10 scaffold (b). In both cases, the selected pore measured about 300 μm on the transversal plane, but on the sagittal plane, it was inferior to 100 μm (a) and 200 μm (b). Figure S2: Sagittal cut of MicroCT image reconstruction of a PCL-BGMS10 scaffold (a) in gray-scale (PCL low, BGMS10 high gray levels) and (b) illustrating concentric ROIs: (i) 5 μm thick surface, i.e., outmost shell (blue); (ii) 5 μm thick outer shell (light blue); (iii) 25 μm thick mid shell (green); (iv) 50 μm thick inner shell (yellow); (v) 100 μm thick inner shell (orange); (vi) core (red).

## Abbreviations

AFM	Atomic Force Microscopy
ALP	Alkaline Phosphatase
ATR	Attenuated Total Reflection
BG	Bioactive glass
BM-MSCs	Bone marrow mesenchymal stem cells
BSP	Bone Sialoprotein
ECM	Extracellular Matrix
EDX	Energy-dispersive X-ray analysis
FTIR	Fourier-Transform Infrared Spectrophotometry
GAPDH	Glyceraldehyde-3-phosphate dehydrogenase
GPU	Graphics Processing Unit
k	Cantilever Stiffness
MicroCT	Micro-Computed Tomography
MSCs	Mesenchymal Stem Cells
OC	Osteocalcin
OP	Osteopontin
OSX	Transcription factor Osterix
R	Curvature radius of the AFM spherical tip
ROI	Region of interest
RUNX	RUNX family transcription factor 2
SEM	Scanning electron microscopy
PCL	Polycaprolactone
2D	Two-dimensional
3D	Three-dimensional
