# Supplementary material for: A Multidisciplinary Evaluation of Three-Dimensional Polycaprolactone Bioactive Glass Scaffolds for Bone Tissue Engineering Purposes
Source: Materials (Basel). 2024 May 17;17(10):2413. doi: 10.3390/ma17102413 (PMC11122918; doi:10.3390/ma17102413)
Supplement: Supplementary file 1 [file materials-17-02413-s001.zip › materials-2964524-supplementary.pdf]

Supplementary Materials for the manuscript entitled “**A Multidisciplinary Evaluation of Three Dimensional Poly-caprolactone Bioactive Glass Scaffolds for Bone-Tissue Engineering Purposes”**

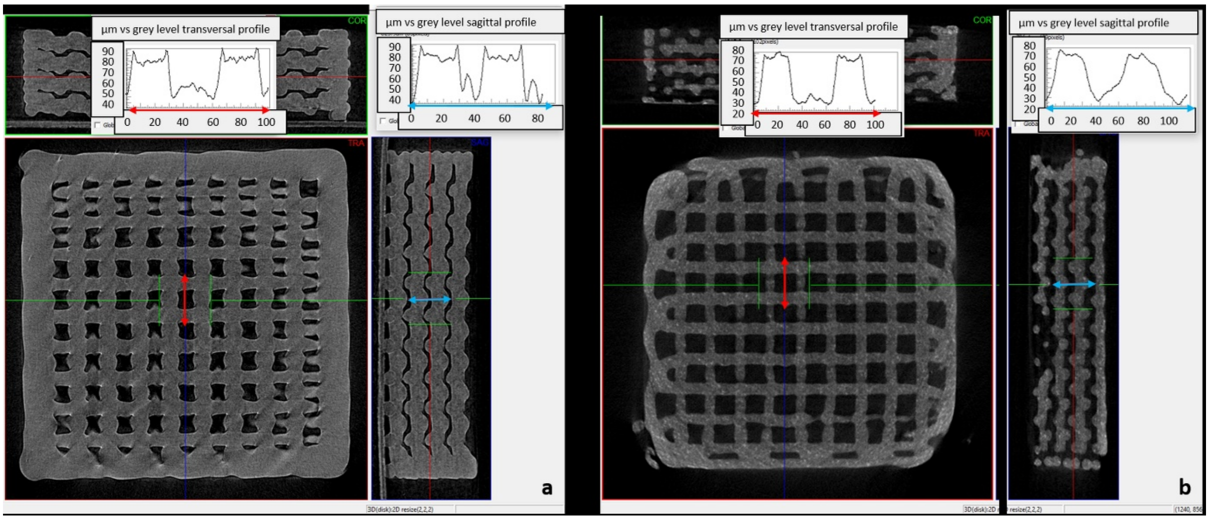

Figure S1. Cutting planes of the microCT image reconstruction of a PCL scaffold (a) and of a PCL-BGMS10 scaffold (b). In both cases the selected pore measured about 300 µm on the transversal plane, but on the sagittal plane it was inferior to 100 µm (a) and 200 µm (b)

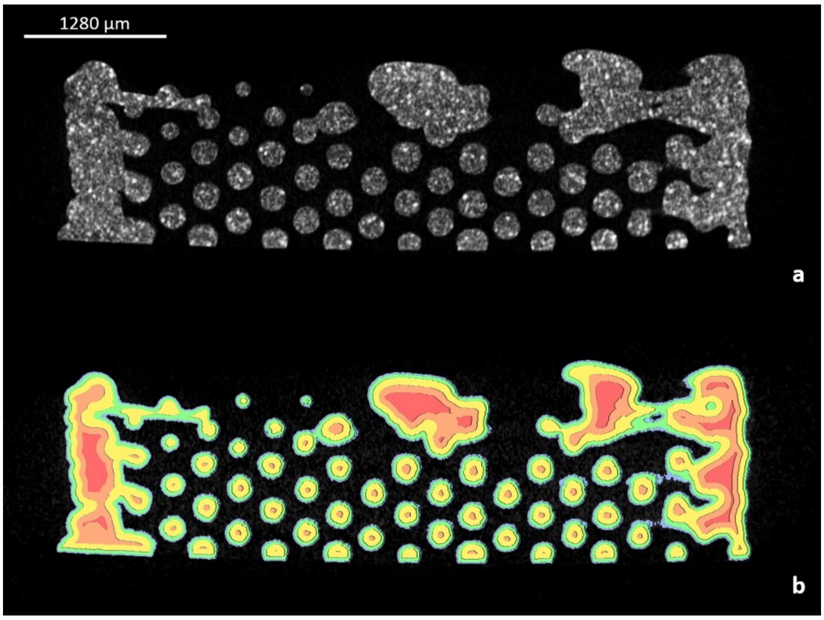

Figure S2. Sagittal cut of microCT image reconstruction of a PCL- BGMS10 scaffold (a) in gray-scale (PCL low, BGMS10 high gray levels) and (b) illustrating concentric ROIs: (i) 5 µm thick surface, i.e. outest, shell (blue); (ii) 5 µm thick outer shell (light blue); (iii) 25 µm thick mid shell (green); (iv) 50 µm thick inner shell (yellow); (v) 100 µm thick innest shell (orange); (vi) core (red).
